# Intravascular large B‐cell lymphoma presenting as Richter's syndrome with cerebral involvement in a patient with chronic lymphocytic leukemia

**DOI:** 10.1002/ccr3.1087

**Published:** 2017-07-20

**Authors:** Robert Puckrin, Paula Pop, Zeina Ghorab, Julia Keith, Lisa Chodirker, Yulia Lin, Jeannie Callum

**Affiliations:** ^1^ Postgraduate Medical Education University of Toronto Toronto Ontario Canada; ^2^ Department of Anatomic Pathology Sunnybrook Health Sciences Centre Toronto Ontario Canada; ^3^ Department of Hematology Sunnybrook Health Sciences Centre Toronto Ontario Canada; ^4^ Department of Clinical Pathology Sunnybrook Health Sciences Centre Toronto Ontario Canada

**Keywords:** Chronic lymphocytic leukemia, intravascular large B‐cell lymphoma, meningeal lymphomatosis, Richter's transformation

## Abstract

Intravascular large B‐cell lymphoma (IVLBCL) is an aggressive non‐Hodgkin's lymphoma which can present with B symptoms, rash, and neurological deterioration. Up to 10% of cases of IVLBCL are associated with other hematological neoplasms, including this extremely rare presentation of IVLBCL as Richter's transformation in chronic lymphocytic leukemia.

## Introduction

Intravascular large B‐cell lymphoma (IVLBCL) is a rare type of non‐Hodgkin's lymphoma associated with intravascular proliferation of malignant cells and diverse clinical manifestations. We report the case of a 76‐year‐old man with a 6 year history of chronic lymphocytic leukemia (CLL), Rai stage 0, who presented with B symptoms, functional decline, and neurological deterioration. Investigations revealed new splenomegaly, cytopenias, elevated LDH, and pachymeningeal thickening on cerebral MRI. There was evidence of CLL on flow cytometry of peripheral blood and bone marrow. Meningeal biopsy ultimately revealed the diagnosis of IVLBCL, which was confirmed to be clonally related to the underlying CLL by polymerase chain reaction (PCR) of the immunoglobulin heavy chain (IgVH) gene. This is an extremely rare case of IVLBCL arising as Richter's transformation in CLL. We review the literature on IVLBCL, with focus on the neurological manifestations of this disease, its coexistence with other hematological neoplasms, and the need for further research to guide treatment of IVLBCL with cerebral involvement.

## Case Report

A 76‐year‐old man with a 6 year history of untreated chronic lymphocytic leukemia (CLL), Rai stage 0, was referred to our Hematology service with a 2 week history of fevers, night sweats, productive cough, and marked functional decline. During his admission to hospital, he developed progressive neurological deterioration with confusion, generalized weakness, expressive aphasia, and brief tonic–clonic seizures.

On physical examination, the patient was hemodynamically stable but developed periodic fevers >39°C and mild hypoxemia requiring supplemental oxygen via nasal cannula. There was no palpable peripheral lymphadenopathy, although there was dullness to percussion over Traube's space. A violaceous, nonindurated 5 by 6 cm patch was observed on the posterior neck. As the patient's neurological status declined, he was noted to have expressive language deficits. The remainder of the physical examination was unremarkable.

The patient's past medical history also included amaurosis fugax, mild Alzheimer's type dementia, remote traumatic brain injury and subarachnoid hemorrhage, and benign prostatic hyperplasia. His current medications included acetylsalicylic acid, tamsulosin, donepezil, and risperidone. Baseline Eastern Cooperative Oncology Group (ECOG) performance status was 0.

Laboratory investigations revealed new cytopenias including a normocytic anemia with hemoglobin (Hb) 117 g/L and thrombocytopenia with platelet count 78,000/mm^3^. White blood cell (WBC) count was 6400/mm^3^ with an absolute lymphocyte count (ALC) of 3500/mm^3^, decreased from baseline ALC of 10,000/mm^3^ 4 months prior to presentation. Smudge cells and small mature lymphocytes were identified on blood smear review. LDH was strikingly elevated at 1322 U/L, and inflammatory markers were raised with CRP 78 mg/L and ESR 25 mm/h. Other markers of cell turnover including uric acid, potassium, phosphorus, and calcium were normal. Serum albumin was 27 g/L without significant proteinuria. Serum creatinine, thyroid‐stimulating hormone, and liver enzymes were within the normal ranges. There was no evidence of hemolysis or disseminated intravascular coagulation with normal reticulocyte count, haptoglobin, bilirubin, prothrombin time (PT), partial thromboplastin time (PTT), and fibrinogen levels. There was no monoclonal protein on serum protein electrophoresis, and quantitative immunoglobulin testing revealed moderate reductions in levels of IgA (0.67 g/L), IgG (4.15 g/L), and IgM (0.33 g/L). An infectious work‐up including cultures of blood, urine, and cerebrospinal fluid (CSF) as well as serologies for human immunodeficiency virus (HIV), hepatitis B virus, hepatitis C virus, West Nile virus, syphilis, and cryptococcus were all negative.

Peripheral blood flow cytometry was in keeping with the patient's known CLL, with a population of lymphoid cells expressing B‐cell markers CD19, dim CD20, dim CD22, CD23, and dim Cd11c with aberrant CD5 coexpression and dim kappa light chain restriction. Identical findings were obtained on flow cytometry of a bone marrow aspirate and biopsy, with no evidence of marrow involvement by hemophagocytosis or transformed lymphoma.

**Figure 1:**
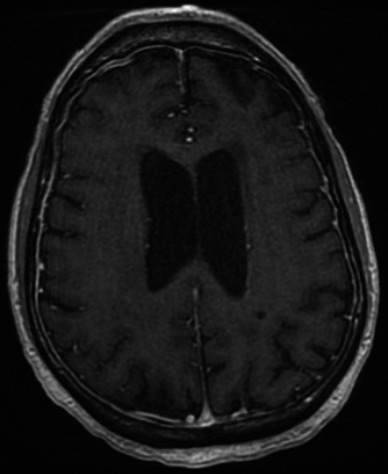
Axial image from MRI of the brain demonstrating generalized smooth pachymeningeal thickening and enhancement secondary to lymphomatous infiltration.

Computed tomography (CT) scans of the head, chest, abdomen, and pelvis did not identify any lymphadenopathy, but did reveal new splenomegaly measuring up to 16 cm containing several foci of low attenuation. There were also bilateral ground‐glass opacities and interlobular septal thickening within the mid and lower lung zones, as well as small pleural effusions, diffuse body wall edema, and moderate amounts of pelvic free fluid. Echocardiogram revealed normal cardiac function and no pericardial effusion. Magnetic resonance imaging (MRI) of the brain and spine was notable for generalized smooth pachymeningeal thickening and enhancement along the bilateral cerebral surfaces, suggestive of an infectious or lymphomatous process (Fig. 1). There were no central nervous system (CNS) parenchymal lesions. Initial CSF studies revealed normal glucose and protein levels and 0 RBCs/mm^3^ and 4 WBCs/mm^3^; cytology slides revealed no cellular elements. Repeat large volume CSF sampling again demonstrated normal glucose and protein levels with 108 RBCs/mm^3^ and 3 WBCs/mm^3^. CSF cytology was significant for the presence of rare abnormal large lymphoid cells, but there were insufficient cells to perform flow cytometry.

**Figure 2:**
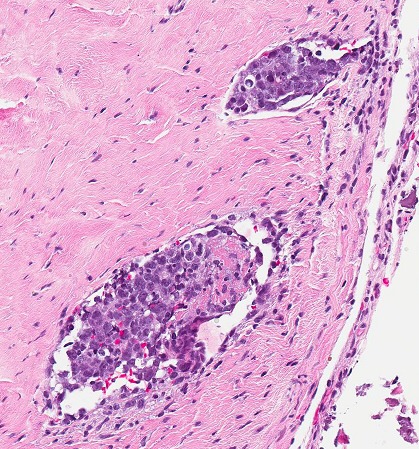
Hematoxylin and eosin staining demonstrating meningeal blood vessels distended by intravascular collections of malignant lymphocytes.

**Figure 3:**
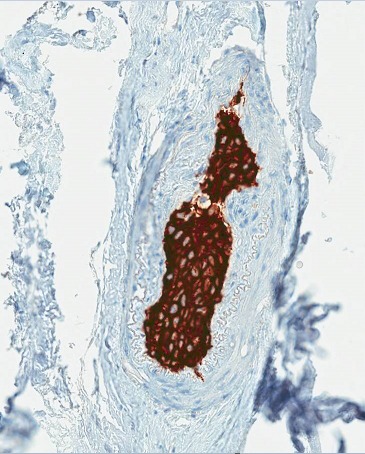
Immunohistochemistry staining demonstrating strong expression of CD20 by IVLBCL cells.

Given the strong suspicion for Richter's transformation with lymphomatous pachymeningeal infiltration, a frontal craniotomy and dural biopsy were performed which revealed blood vessels distended by intravascular collections of large atypical cells containing minimal cytoplasm and large atypical nuclei (Fig. 2). These tumor cells demonstrated strong expression of CD20 (Fig. 3), CD5, and MUM1 with dim expression of CD10 and bcl6. In situ hybridization was negative for Epstein–Barr virus. Proliferative activity was markedly elevated with Ki67 90%. These findings were diagnostic of meningeal infiltration with intravascular large B‐cell lymphoma (IVBCL). A clonal relationship to the underlying CLL was confirmed by polymerase chain reaction (PCR) of the immunoglobulin heavy chain (IgVH) gene.

An empiric trial of oral dexamethasone resulted in resolution of the neck rash and improvement in platelet count and mental status. The patient was treated with six cycles of rituximab, cyclophosphamide, doxorubicin, vincristine, and prednisone (R‐CHOP) with CNS‐directed therapy with high‐dose intravenous and intrathecal methotrexate. Seven months after his initial presentation, the patient has completed treatment with improvement of his peripheral cell counts (Hb 115 g/L, WBC 5.5/mm^3^, and platelets 144/mm^3^) and LDH (158 U/L), and near complete resolution of the splenomegaly and meningeal thickening on follow‐up imaging. Although he does have ongoing cognitive impairment, his functional status and aphasia have significantly improved with treatment.

## Discussion

Intravascular large B‐cell lymphoma (IVLBCL) is a rare and aggressive extranodal non‐Hodgkin's lymphoma characterized by proliferation of neoplastic cells within the lumen of small‐ to medium‐sized blood vessels 1, 2, 3, 4. Recognized as a distinct type of mature B‐cell lymphoma in the 2016 World Health Organization classification 5, IVLBCL primarily affects the elderly (median age 64 years) with an estimated incidence of 0.5–1 case per 1 million 6, 7, 8. Three different clinical phenotypes of IVLBCL have been described 1. Classical IVLBCL commonly presents in Western countries with B symptoms, neurological deterioration, and rashes, while hemophagocytosis‐related IVLBCL is more common in Japan and is characterized by fever, cytopenias, hepatosplenomegaly, and bone marrow involvement with the hemophagocytic syndrome 9. Finally, the cutaneous variant of IVLBCL primarily affects young females and is distinguished by isolated dermatologic involvement and a more favorable prognosis 10. IVLBCL can present with a myriad of other signs and symptoms reflecting the underlying organ of involvement, including myocardial infarction 11, cholecystitis 12, and glomerulonephritis 13. As in the case of our patient, respiratory involvement is common in IVLBCL 14, and pleural effusions occur in up to 40% of cases and may be a negative prognostic marker 15. Laboratory findings of IVLBCL are nonspecific but frequently include elevated LDH (86%), elevated *β*2 microglobulin (82%), anemia (63%), elevated ESR (43%), thrombocytopenia (29%), leukopenia (24%), hypoalbuminemia (18%), and a monoclonal serum protein (14%) 1, 8. IVLBCL is notoriously difficult to detect due to the sequestration of malignant cells in capillaries and postcapillary venules 1. As in the present case, relatively few patients have evidence of IVLBCL involving the peripheral blood (5–9%) 1, lymph nodes (4%) 8, or bone marrow (30%) 9. Tissue biopsy of an affected organ is thus key to the diagnosis of IVLBCL, although there may also be a role for random skin biopsies from seemingly unaffected skin sites in the detection of disease 8.

The predominantly neurological symptomatology of the present case reflects the predilection for CNS involvement in intravascular lymphoma, which occurs in over 40% of affected patients 7. The most common CNS manifestations are rapidly progressive cognitive impairment (61%), motor weakness (22%), and epileptic seizures (13%). Numerous other presentations have been reported including visual complaints, ataxia, headache, myelopathy, dysarthria, cranial nerve deficits, and stroke‐like presentations 7. CSF analysis is typically nondiagnostic of intravascular lymphoma of the CNS; although CSF pleocytosis is present in 90% of patients and elevated protein levels in 57%, cytology is usually negative for malignant cells 16. Cerebral MRI findings are varied and nonspecific, and can mimic other conditions such as CNS vasculitis with multifocal sites of infarction and nonspecific white matter lesions. Less common radiographic abnormalities include intraparenchymal mass‐like lesions or, as in the case of our patient, isolated meningeal enhancement 17, 18. Neuroimaging studies may be falsely negative in up to half of affected patients 10, leading to growing interest in the use of positron emission tomography (PET) to localize metabolically active sites of IVLBCL 19. The diagnosis of intravascular lymphoma thus requires a high index of suspicion, as exemplified by the nonspecific neurological symptoms of our patient, lack of diagnostic findings on peripheral blood and bone marrow studies, and the initially unremarkable CT of the head and CSF analysis. This highlights the importance of cerebral MRI, repeat large volume CSF cytology, and invasive tissue sampling when a diagnosis of IVLBCL is suspected.

This presentation of intravascular lymphoma arising as Richter's syndrome in the setting of previously stable CLL is extremely rare. To the best of our knowledge, there is only one previous report of IVLBCL associated with CLL 20. Richter's syndrome occurs in 2–8% of patients with CLL, typically manifesting as de novo or transformed diffuse large B‐cell lymphoma (DLBCL) 21. Numerous case reports have documented the synchronous or metachronous occurrence of IVLBCL with other neoplasms, including benign tumors and both solid and hematological malignancies 10. Up to 10% of cases of intravascular lymphoma occur in patients with previous non‐Hodgkin's lymphoma, most commonly morphologically similar cases of aggressive DLBCL 14. However, IVLBCL has also occurred in patients with acute lymphoblastic leukemia 22, follicular lymphoma 23, marginal zone lymphoma 10, ALK‐positive anaplastic large cell lymphoma 24, Waldenstrom's macroglobulinemia 25, and solitary plasmacytoma 26. It is unknown if most cases of IVLBCL arise clonally or independently from the coexistent lymphoma, although a clonal relationship has been established using PCR of the immunoglobulin heavy chain (IgVH) gene in at least four cases of DLBCL associated with IVLBCL 27, 28, 29, 30. The genetic confirmation of a clonal relationship between CLL and IVLBCL in this case provides further evidence that IVLBCL can arise as a transformed lymphoma from a variety of hematological malignancies.

Previous studies demonstrate that 80% of cases of IVLBCL arise from a nongerminal center (non‐GCB) cell of origin, as confirmed by immunophenotyping and IgVH variable region gene analyses 8, 31, 32. In contrast, the case presented here expresses germinal cell markers CD10 and bcl6, suggesting it is of germinal center (GCB) origin as classified by Han's algorithm 33. Although determination of cell of origin has significant prognostic implications in DLBCL, this was not confirmed in a large cohort of patients with intravascular lymphoma 34. Additionally, this IVLBCL demonstrated strong expression of CD5, a surface marker found in only 9% of intravascular lymphomas 6, suggesting a possible link between the frequent expression of CD5 in CLL and transformation into IVLBCL. Expression of CD5 in intravascular lymphoma does not appear to impact overall survival, although it may be associated with less frequent neurological manifestations and a higher prevalence of thrombocytopenia and peripheral blood and bone marrow involvement than CD5‐negative IVLBCL 34. Further research is therefore required to clarify the prognostic significance of different immunohistochemistry findings in intravascular lymphoma.

Treatment of IVLBCL remains difficult and associated with poor outcomes, with a median survival of 5 months from time of symptom onset 6. Poor prognostic factors include age over 70 years old, CNS involvement, and LDH >700 U/L 6. The nonspecific clinical findings in IVLBCL often lead to delays in diagnosis, and patients typically present with disseminated disease requiring systemic treatment 6, 8. Cornerstone therapy consists of anthracycline‐based regimens such as CHOP, which leads to a 3‐year overall survival of 30% 1. The addition of rituximab to CHOP has significantly improved 3‐year overall survival up to 60% 2, and doubled the median time from treatment to death compared to nonrituximab‐based regimens (36 vs. 15 months, *P ≤ *0.0001) 6. However, there are no published randomised trials in the treatment of IVLBCL, so estimates of treatment efficacy are based on retrospective case reports and limited by small study sizes, short follow‐up times, and risk of publication bias 35. Furthermore, the optimal management of IVLBCL with CNS involvement remains uncertain 2. Many experts recommend the use of drugs with greater CNS penetrance, such as high‐dose systemic methotrexate or cytarabine 1, given the evidence for efficacy in primary CNS lymphoma 36. However, others have suggested that CNS‐directed therapy can be omitted because the intravascular location of malignant lymphocytes may not require the administration of drugs that penetrate the blood brain barrier 37. Methotrexate appears to be utilized in as few as 4% of cases of IVLBCL 6, and the largest retrospective analysis to date of 276 patients with cerebral IVLBCL did not identify a survival benefit with methotrexate compared to nonmethotrexate‐based regimens (16.0 vs. 20.0 months, *P = *0.15) 7. Further research is therefore warranted to ascertain the degree of brain parenchymal infiltration in CNS intravascular lymphoma, and to determine if these patients benefit from CNS‐directed therapy with methotrexate in addition to R‐CHOP 6. Finally, there are rare reports of positive outcomes associated with autologous stem cell transplantation in IVLBCL, although this treatment is unavailable to most patients due to their advanced age and poor performance status 2.

## Conclusion

Intravascular large B‐cell lymphoma (IVLBCL) is a rare and aggressive non‐Hodgkin's lymphoma characterized by the intravascular proliferation of malignant lymphocytes. Clinical manifestations include B symptoms, functional decline, rash, and CNS involvement in over 40% of cases. Diagnosis often requires a high index of suspicion and invasive tissue sampling. Up to 10% of cases of IVLBCL arise clonally or independently from other hematological malignancies, although this presentation of IVLBCL as Richter's transformation in CLL is extremely rare. Standard treatment for IVLBCL consists of R‐CHOP, and the role for CNS‐directed therapy with methotrexate in patients with cerebral involvement requires further investigation.

## Authorship

RP: performed the literature review and wrote the manuscript. PP: wrote the manuscript. ZG, JK, LC, and YL: reviewed and edited the manuscript for important intellectual content. JC: supervised the research process and reviewed and edited the manuscript for important intellectual content.

## Disclosures

Informed consent was obtained from the patient prior to publication of this case report. This case report received no funding from any source.

## Conflict of Interests

The authors have no conflict of interests to declare.
